# Generalized linear mixed quantile regression with panel data

**DOI:** 10.1371/journal.pone.0237326

**Published:** 2020-08-11

**Authors:** Xiaoming Lu, Zhaozhi Fan

**Affiliations:** Department of Mathematics and Statistics, Memorial University of Newfoundland, Newfoundland, Canada; Cleveland Clinic Lerner Research Institute, UNITED STATES

## Abstract

A new generalized linear mixed quantile model for panel data is proposed. This proposed approach applies GEE with smoothed estimating functions, which leads to asymptotically equivalent estimation of the regression coefficients. Random effects are predicted by using the best linear unbiased predictors (BLUP) based on the Tweedie exponential dispersion distributions which cover a wide range of distributions, including those widely used ones, such as the normal distribution, Poisson distribution and gamma distribution. A Taylor expansion of the quantile estimating function is used to linearize the random effects in the quantile process. The parameter estimation is based on the Newton-Raphson iteration method. Our proposed quantile mixed model gives consistent estimates that have asymptotic normal distributions. Simulation studies are carried out to investigate the small sample performance of the proposed approach. As an illustration, the proposed method is applied to analyze the epilepsy data.

## Introduction

Panel data and other repeated measurements are very common in clinical tests of new drugs, economic studies, as well as many other areas of applied studies. Such data are from the subjects measured repeatedly over time. In the collection of data, correlation may exist between repeated measurements within the same subject. Furthermore, data collected from biological units often exhibit over-dispersion, heteroscedasticity and within-subject dependence, such as the epileptic seizure count data. [1] In this case, both the subject- and observation-level dependence should be accounted when constructing an analysis method. Mixed models are widely used in data analysis to account for these issues by introducing random effects. In the literature, mixed regression methods are developed to evaluate covariate effects on the mean of a response variable. [2–4] The new anti-epileptic drug progabide may have different effects for patients with different seizure rates. [1] Often, subjects at different levels of a response variable might be affected by risk factors differently or event in opposite direction. The traditional regression method modelling the conditional mean may not be informative enough to catch these effects and could even provide misleading results on the effects of covariates. Quantile regression would be more appealing for the analysis of data with such heteroscedasticity.

Quantile regression has become a widely used technique in statistical studies and applications. [5] In stead of modelling the effects of covariates on the conditional mean, quantile regression models are based on the conditional quantiles which extends regression for the mean to the analysis of the entire conditional distribution of the outcome variable. Therefore, location, scale and shape of the distribution can be fully examined to provide a complete view of how the covariates affect the entire response distribution. Comparing to the classical mean regression, quantile regression is more robust to outliers and also invariant to monotonic transformations. Quantile regressions do not require any Gaussian assumptions for the response and can deal with heavy-tailed and asymmetric data.

The extension of quantile regression (QR) to repeated measures has been a rising area of research in statistics. A quasi-likelihood method firstly proposed with estimated correlation matrix in the median regression. [6] This method was extended to construct a weighted GEE model. [7] A general stationary auto-correlation structure for the covariance matrix was applied in a proposed weighted quantile regression model. [8]

Mixed effects regression models are very popular in analysing clustered, repeated measurements and panel data, which are collected from clinical trials, agricultural field studies, environmental and wildlife ecology studies, to name a few. Literature contributions have extended QR into mixed modelling framework. A quantile random effects model with a penalized likelihood was developed. [9] Using the asymmetric Laplace distribution (ALD), a method was proposed to account for the within-subject correlations using a random intercept. [10] A linear mixed quantile regression model by incorporating a multivariate Laplace distribution was suggested. [11] A linear quantile mixed model which extends quantile regression models (QR-LMM) was developed with random intercepts to include random slopes. [12] Then an extension of QR-LMM was provided to modelling and estimation of nonlinear quantile regression when data are clustered within two-level nested designs. [13] The induced smoothing method has been extended to quantile regression to redefine smoothed objective functions. [8, 14–16] Literature applied the Newton-Raphson iteration method to eliminate computational issues and automatically get both the estimates of parameters and their covariance matrix.

In this paper, we develop a generalized mixed quantile regression model (1.1) Based on the assumption of Tweedie exponential dispersion distributions, subject-specific and observation-specific random effects are predicted by their orthodox best liner unbiased predictors (BLUP). General stationary auto-correlation matrix has been used to avoid the need to specify any particular working correlation structure between repeated measurements. Continuous quantile estimating functions are obtained by applying the induced smoothing method. A Taylor expansion of estimating functions accommodates the variation of random effects within the estimation of parameters in the quantile regression. Parameters are estimated through Newton-Raphson iterations. The simulation results reveal that our proposed quantile mixed regression model performs well. Parameter estimation obtained by our proposed method is consistent and asymptotically normal.

Our methodology is based on the fact that the mean and a quantile of a positive value (*U*) times a variable *Y*, denoted as *E*(U*Y) and *Q*(U*Y), are equal to the positive value *U* times the mean and quantile of *Y*. That is *E*(U*Y) = U*E(*Y*) and *Q*(U*Y) = U*Q(*Y*). For the same data, the mean and quantile regression analysis can share some common heterogeneity that are measured by random effects. Therefore, we can borrow the random effects from the mean regression when we conduct quantile regression analysis. Because the good properties of Tweedie distributions, we can predict the random effects using their BLUPs. This avoids the integration over random effects and possible sampling step (e.g. Monte Carlo) for the estimation process. Also, it allows us to utilize the simple Newton-Raphson algorithm to update the estimators with much less computation cost than other approaches involving re-sampling stochastic processes (e.g. Bayesian using Markov Chain Monte Carlo).

Currently we are working on a hierarchical model on the two levels of random effects. Results will be reported in a separate paper.

The remainder of this paper proceeds as follows: In the next, we develop the proposed generalized quantile mixed regression method and the iteration steps of parameter estimation. Asymptotic properties are then discussed. To illustrate the performance of the proposed method, we apply the method to the epileptic seizure count data and carry out extensive simulation studies. This paper is concluded with a few remarks in the final section.

## Proposed quantile regression models

In this section, we first introduce a class of Tweedie exponential dispersion distributions. The good properties of the Tweedie distributions can be applied to predict random effects in our following proposed mixed models. If random variable *Y* is said to follow a Tweedie exponential dispersion distribution with location parameter *μ*, dispersion parameters *σ*^2^ and another shape parameter *q*, denoted as *Tw*_*q*_(*μ*, *σ*^2^), its density is of the form

$$\begin{array}{c} f_{q}\left(y;\mu ,\sigma^{2}\right)=\{\begin{array}{cc} c_{q}\left(y;\sigma^{2}\right)exp\{\frac{1}{\sigma^{2}}(\frac{y\mu^{1-q}}{1-q}-\frac{\mu^{2-q}}{2-q})\} & \;\text{if}\;q\neq 1,2,\\ c_{2}\left(y;\sigma^{2}\right)exp\{-\frac{1}{\sigma^{2}}(\frac{y}{\mu }+\log\left(\mu \right))\} & \;\text{if}\;q=2,\\ c_{1}\left(y\right)exp\{y\log(\mu )-\mu \} & \;\text{if}\;q=1, \end{array} \end{array}$$ (1)

where *c*_*q*_(*y*;*σ*^2^) are given. [17] Also, *E*(*Y*) = *μ* and *Var*(*Y*) = *σ*^2^ *μ*^*q*^ are the mean and variance of the distribution. This Tweedie family actually covers many well-known distributions which gives us the flexibility to use it for the assumptions of random effects in the generalized linear mixed models. Specifically, it is normal when *q* = 0, Poisson when *q* = 1 and *σ*^2^ = 1, gamma when *q* = 2, and inverse-Gaussian when *q* = 3.

### Data setup and the models

In a repeated measurement setup, responses are collected along with certain multidimensional covariates from a large number of independent individuals. Let $y_{i1},\ldots ,y_{ij},\ldots ,y_{in_{i}}$ be *n*_*i*_ ≥ 2 repeated measures observed from the *i*th subject, for *i* = 1, …, *m*, where *m* is a positive integer. Motivated by the epileptic seizure count data which exhibits a high degree of over-dispersion, we assume that there exist subject-specific and observation-specific random effects. Let *U* = (U_*_, U_**_) where U_*_ = (*U*_1_, …, *U*_*m*_)^*T*^ and $U_{**}={\left(U_{11},\ldots ,U_{1n_{1}},\ldots ,U_{mn_{m}}\right)}^{T}$ denote the vectors of subject-specific and observation-specific random effects, respectively. The vector *x*_*ij*_ = (*x*_*ij*1_, …, *x*_*ijp*_)^*T*^ is for the *p*-dimensional covariate vector corresponding to *y*_*ij*_.

Assume that the subject-specific random effects *U*_1_, …, *U*_*m*_ are positive, independent and identically distributed with mean *E*(*U*_*i*_) = 1 (log(*E*(*U*_*i*_) = 0), later we assume the conditional mean of the response is *u*_*ij*_ *μ*_*ij*_) and variance var(*U*_*i*_) = *σ*^2^. Given U_*_ = u_*_ = (*u*_1_, …, *u*_*m*_)^*T*^, the observation-specific random effects $U_{11},\ldots ,U_{1n_{1}},\ldots ,U_{mn_{m}}$ are positive and conditionally independent. The distributions of these random effects are members of the Tweedie family: *U*_*i*_ ∼ *Tw*_*r*_(1, *σ*^2^), *r* ≥ 2 and $U_{ij}|U_{i}=u_{i}∼Tw_{t}\left(u_{i},\nu^{2}u_{i}^{1-t}\right)$, *t* ≥ 2. When *ν*^2^ = 0, *U*_*ij*_ = *U*_*i*_ for all *j* = 1, …, *n*_*i*_, we have just one level of random effects in our mixed model. This hierarchical structure of random effects provides us with additional flexibility when modelling the baseline variation, as well as correlation structure among repeated observations.

Assume that the conditional distribution of *Y*_*ij*_, given *U* = *u*, depends on *u*_*ij*_,

$$\begin{array}{c} Y_{ij}|U=u∼Tw_{q}\left(u_{ij}\mu_{ij},\gamma^{2}u_{ij}^{1-q}\right), \end{array}$$

where $\mu_{ij}=\exp\left(x_{ij}^{T}\beta \right)$; *β* is the vector of regression parameters; and *γ*^2^ is a dispersion parameter. This assumption on *Y* is for predicting random effects in the next section but may not be hold for estimating quantile regression parameters. When *q* = 1, the conditional distribution of *Y*_*ij*_ becomes a Poisson distribution with conditional mean *E*(*Y*_*ij*_|*U*) = *u*_*ij*_ *μ*_*ij*_. This gives us the ability to deal with count observations while maintains the flexibility of our model to be applied to analysis of other types of data (i.e. continuous, binary, and ordinal etc.)

The analysis of epileptic data, based on the assumptions of the conditional mean, reveals that the new drug may have different effects for patients with different seizure rates. [1] And the data has a few outliers. To provide a richer characterization of the data, similar assumptions can be made on the conditional quantiles of the response distribution. For a given *U*, the *τ*th quantile of the conditional distribution of *Y*_*ij*_ can be assumed as

$$\begin{array}{c} Q_{\tau }\left(Y_{ij}|U\right)=u_{ij}\mu_{ij}^{\tau }, \end{array}$$ (2)

where $\mu_{ij}^{\tau }=\exp\left(x_{ij}^{T}\beta_{\tau }\right)$ and *β*_*τ*_ is the quantile regression parameter vector. We are interested in consistently estimating *β*_*τ*_ as efficiently as possible.

Note that, model (1) can be rewritten as

$$\begin{array}{c} Q_{\tau }\left(Y_{ij}|U\right)=\exp\left(\log u_{ij}+x_{ij}^{T}\beta_{\tau }\right), \end{array}$$ (3)

which we call it a generalized linear mixed quantile model. When the conditional distribution of the the response is Poisson, the model will be a Poisson linear mixed quantile regression model.

### Orthodox best linear unbiased predictors of random effects

To predict the subject-specific and observation-specific random effects, we introduce an orthodox best linear unbiased predictor (BLUP). [18] That is

$$\begin{array}{c} \hat{U}=E\left(U\right)+\text{cov}\left(U,Y\right){\text{var}}^{-1}\left(Y\right)\left\{Y-E\left(Y\right)\right\}. \end{array}$$

Using properties of Tweedie distributions, the subject-specific random effects are predicted by

$$\begin{array}{c} {\hat{U}}_{i}=\frac{1+\sigma^{2}\sum_{j=1}^{n_{i}}w_{ij}{\mu_{ij}}^{1-q}Y_{ij}}{1+\sigma^{2}\sum_{j=1}^{ni}w_{ij}{\mu_{ij}}^{2-q}}, \end{array}$$ (4)

where

$$\begin{array}{c} w_{ij}=\frac{1}{\gamma^{2}+\nu^{2}{\mu_{ij}}^{2-q}}. \end{array}$$

The observation-specific random effects are predicted similarly as

$$\begin{array}{c} {\hat{U}}_{ij}=\gamma^{2}w_{ij}{\hat{U}}_{i}+\nu^{2}w_{ij}{\mu_{ij}}^{1-q}Y_{ij}. \end{array}$$ (5)

The mean squared distances between these random effects and their predictors are

$$\begin{array}{c} c_{i}=E{\left({\hat{U}}_{i}-U_{i}\right)}^{2}=\frac{\sigma^{2}}{1+\sigma^{2}\sum_{j=1}^{n_{i}}w_{ij}{\mu_{ij}}^{2-q}} \end{array}$$ (6)

and

$$\begin{array}{c} c_{ij}=E{\left({\hat{U}}_{ij}-U_{ij}\right)}^{2}=\gamma^{2}w_{ij}\left(\nu^{2}+\gamma^{2}c_{i}w_{ij}\right). \end{array}$$ (7)

Based on the mean squared distances, consistency results about random effects predictors can be drawn under ‘small dispersion asymptotic’ (${\hat{U}}_{i}\overset{p}{\to }U_{i}$ as *σ*^2^ → 0, ${\hat{U}}_{ij}\overset{p}{\to }U_{ij}$ as *σ*^2^+ *ν*^2^ → 0) and large sample asymptotic (${\hat{U}}_{i}\overset{p}{\to }U_{i}$ as *n*_*i*_ → ∞). See [19] for proofs.

### Prediction

Using the BLUPs of the random effects, ${\hat{U}}_{ij}$, the estimating equation is built as the following:

$$\begin{array}{c} \Psi^{\prime }\left(\beta \right)=\sum_{i=1}^{m}\sum_{j=1}^{n_{i}}x_{ij}\frac{{\mu_{ij}}^{1-q}}{\gamma^{2}}\left\{y_{ij}-{\hat{U}}_{ij}\mu_{ij}\right\}=0. \end{array}$$ (8)

For the unknown parameters *σ*^2^, *ν*^2^ and *γ*^2^, we apply the following adjusted Pearson estimators

$$\begin{array}{c} {\hat{\sigma }}^{2}=\frac{1}{m}\sum_{i=1}^{m}{\left({\hat{U}}_{i}-1\right)}^{2}+\frac{1}{m}\sum_{i=1}^{m}c_{i}, \end{array}$$

$$\begin{array}{c} {\hat{\nu }}^{2}=\frac{1}{\sum_{i=1}^{m}n_{i}}\sum_{i=1}^{m}\sum_{j=1}^{n_{i}}\left\{{\left({\hat{U}}_{ij}-{\hat{U}}_{i}\right)}^{2}+c_{ij}+c_{i}-2\gamma^{2}c_{i}w_{ij}\right\} \end{array}$$

and

$$\begin{array}{c} {\hat{\gamma }}^{2}=\frac{1}{\sum_{i=1}^{m}n_{i}}\sum_{i=1}^{m}\sum_{j=1}^{n_{i}}\{\frac{{\left(y_{ij}-{\hat{U}}_{i}\mu_{ij}\right)}^{2}}{{\mu_{ij}}^{q}}+c_{i}{\mu_{ij}}^{2-q}\}. \end{array}$$

These dispersion estimators are obtained iteratively, with initial values provided in the appendix. They are unbiased, when adjusted by their bias corrections.

To solve the estimating Eq (8), we apply the Newton-Raphson algorithm to update the value of regression parameter *β* by

$$\begin{array}{c} \beta^{*}=\beta -S^{-1}\left(\beta \right)\Psi^{\prime }\left(\beta \right), \end{array}$$

until convergence. And the sensitive matrix *S*(*β*) has the form

$$\begin{array}{c} \begin{array}{cc} S(\beta )= & -\sum_{i=1}^{m}\sum_{j=1}^{n_{i}}\frac{1}{\gamma^{2}}{\mu_{ij}}^{2-q}x_{ij}x_{ij}^{T}+\sum_{i=1}^{m}c_{i}(\sum_{j=1}^{n_{i}}w_{ij}{\mu_{ij}}^{2-q}x_{ij}){\left(\sum_{j=1}^{n_{i}}w_{ij}{\mu_{ij}}^{2-q}x_{ij}\right)}^{T}\\ & +\sum_{i=1}^{m}\sum_{j=1}^{n_{i}}\frac{\nu^{2}w_{ij}}{\gamma^{2}}\left({\mu_{ij}}^{2-q}x_{ij}\right){\left({\mu_{ij}}^{2-q}x_{ij}\right)}^{T}. \end{array} \end{array}$$

Initial values to start the first iteration are deferred to the S1 Appendix. Within each iteration, subject-specific and observation-specific random effects are updated by their BLUPs, ${\hat{U}}_{*}$ and ${\hat{U}}_{**}$ in (4) and (5), at *β* = β^*^. Dispersion parameters are then updated by their adjusted Pearson estimators at *β* = β^*^ using updated ${\hat{U}}_{*}$ and updated ${\hat{U}}_{**}$.

### Estimation of quantile regression parameters

The quantile regression parameter, *β*_*τ*_, can be estimated by minimizing the following objective function, pretending that *U*_*ij*_ are observable,

$$\begin{array}{c} K\left(\beta_{\tau },U\right)=\sum_{i=1}^{m}\sum_{j=1}^{n_{i}}\rho_{\tau }\left(y_{ij}-U_{ij}\mu_{ij}^{\tau }\right), \end{array}$$ (9)

where *ρ*_*τ*_(*s*) = *s*(*τ*−*I*(*s* ≤ 0)) is a check loss function [5], *U*_*ij*_ are unspecified random effects. Estimating equations can be derived from function (9) as follows:

$$\begin{array}{c} \Psi_{0}\left(\beta_{\tau },U\right)=\frac{\partial K(\beta_{\tau },U)}{\partial \beta_{\tau }}=\sum_{i=1}^{m}\sum_{j=1}^{n_{i}}x_{ij}U_{ij}\mu_{ij}^{\tau }\psi_{\tau }\left(y_{ij}-U_{ij}\mu_{ij}^{\tau }\right)=0, \end{array}$$ (10)

where $\psi_{\tau }\left(s\right)=\rho_{\tau }^{\prime }\left(s\right)=\tau -I\left(s<0\right)$ is a discontinuous function.

In order to incorporate the within correlations between repeated measurements in quantile regression models, we denote $\varepsilon_{i}={\left(\varepsilon_{i1},\ldots ,\varepsilon_{ij},\ldots ,\varepsilon_{in_{i}}\right)}^{T}$ which is a continuous error vector with elements $\varepsilon_{ij}=y_{ij}-U_{ij}\mu_{ij}^{\tau }$ satisfying Pr(*ϵ*_*ij*_ ≤ 0) = *τ* with unknown density *f*_*ij*_(⋅). Let $\Gamma_{i}=\text{diag}\left[f_{i1}\left(0\right),\ldots ,f_{in_{i}}\left(0\right)\right]$ and $\Delta_{i}=\text{diag}\left[U_{i1}\mu_{i1}^{\tau },\ldots ,U_{in_{i}}\mu_{in_{i}}^{\tau }\right]$. The term Γ_*i*_ can be well estimated:

$$\begin{array}{c} {\hat{f}}_{ij}\left(0\right)=2h_{n}{\left[\exp\left(x_{ij}^{T}\left({\hat{\beta }}_{\tau +h_{n}}-{\hat{\beta }}_{\tau -h_{n}}\right)\right)\right]}^{-1}, \end{array}$$

where *h*_*n*_ → 0, when *n* → ∞, is a bandwidth parameter. [20] This diagonal matrix describes the dispersions in *ϵ*_*ij*_ and can be simply treated as a scalar matrix when *f*_*ij*_ is difficult to estimate. Since *ψ*_*τ*_(*ϵ*_*i*_) is Bernoulli distributed and can be seen as a random noise vector in the mean regression, we can account for within correlations in quantile regression by estimating the correlation matrix of *ψ*_*τ*_(*ϵ*_*i*_). However, whatever correlation matrix that *ϵ*_*i*_ follows, the correlation matrix of *ψ*_*τ*_(*ϵ*_*i*_) is no longer the same as the one of *ϵ*_*i*_, and it is difficult to specify its correlation structure.

We extend the quasi-likelihood method [6] to quantile regression and apply a general stationary auto-correlation structure for the covariance matrix. Incorporating with random effects, we have the following estimating equation

$$\begin{array}{c} \Psi \left(\beta_{\tau },U\right)=\sum_{i=1}^{m}X_{i}^{T}\Delta_{i}\Gamma_{i}\Sigma_{i}^{-1}\left(\rho \right)\psi_{\tau }\left(\epsilon_{i}\right)=0, \end{array}$$ (11)

where *Σ*_*i*_(*ρ*) is the covariance matrix of *ψ*_*τ*_(*ϵ*_*i*_) with the form $\Sigma_{i}\left(\rho \right)=A_{i}^{\frac{1}{2}}C_{i}\left(\rho \right)A_{i}^{\frac{1}{2}}$ where $A_{i}=\text{diag}\left[\sigma_{i11},\ldots ,\sigma_{in_{i}n_{i}}\right]$ and *σ*_*ijj*_ = (*ψ*_*τ*_(*ϵ*_*ij*_)). The correlation matrix of *ψ*_*τ*_(*ϵ*_*i*_) is denoted as *C*_*i*_(*ρ*) with *ρ* being a correlation parameter. Suppose that the covariance matrix *Σ*_*i*_(*ρ*) has a general stationary auto-correlation structure with the correlation matrix *C*_*i*_(*ρ*) given by

$$\begin{array}{c} C_{i}\left(\rho \right)=(\begin{array}{ccccc} 1 & \rho_{1} & \rho_{2} & \cdots & \rho_{n_{i}-1}\\ \rho_{1} & 1 & \rho_{1} & \cdots & \rho_{n_{i}-2}\\ \vdots & \vdots & \vdots & & \vdots \\ \rho_{n_{i}-1} & \rho_{n_{i}-2} & \rho_{n_{i}-3} & \cdots & 1 \end{array}) \end{array}$$

for all *i* = 1, …, *m*, where the correlation *ρ*_ℓ_ can be estimated by

$$\begin{array}{c} {\hat{\rho }}_{\ell }=\frac{\sum_{i=1}^{m}\sum_{j=1}^{n_{i}-\ell }{\tilde{y}}_{ij}{\tilde{y}}_{i,j+\ell }/\left(m\left(n_{i}-\ell \right)\right)}{\sum_{i=1}^{m}\sum_{j=1}^{n_{i}}{\tilde{y}}_{ij}^{2}/\left(mn_{i}\right)} \end{array}$$

for ℓ = 1, …, *n*_*i*_−1 with ${\tilde{y}}_{ij}$ defined as ${\tilde{y}}_{ij}=\psi_{\tau }(y_{ij}-U_{ij}\mu_{ij}^{\tau })/\sqrt{\sigma_{ijj}}$ At the true parameter *β*_*τ*_, $U_{ij}\mu_{ij}^{\tau }$ is the *τ*th quantile of *y*_*ij*_ conditional on *U*_*ij*_. Hence $\mathrm{Pr}(y_{ij}<U_{ij}\mu_{ij}^{\tau }|U_{ij})=\tau$, which leads to a substitute of *σ*_*ijj*_,

$$\begin{array}{cc} {\tilde{\sigma }}_{ijj} & =\left[\psi_{\tau }\left(\epsilon_{ij}\right)\right]=\left[\tau -I\left(y_{ij}<U_{ij}\mu_{ij}^{\tau }\right)\right]=\left[I\left(y_{ij}<U_{ij}\mu_{ij}^{\tau }\right)\right]\\ & =\left\{E\left[I\left(y_{ij}<U_{ij}\mu_{ij}^{\tau }\right)|U_{ij}\right]\right\}+E\left\{\left[I\left(y_{ij}<U_{ij}\mu_{ij}^{\tau }\right)|U_{ij}\right]\right\}\\ & =\left[\mathrm{Pr}\left(y_{ij}<U_{ij}\mu_{ij}^{\tau }\right)|U_{ij}\right]+E\left[\mathrm{Pr}\left(y_{ij}<U_{ij}\mu_{ij}^{\tau }|U_{ij}\right)\left(1-\mathrm{Pr}\left(y_{ij}<U_{ij}\mu_{ij}^{\tau }|U_{ij}\right)\right)\right]\\ & =\tau (1-\tau ). \end{array}$$

Consequently, the matrix *A*_*i*_ can be replaced with

$$\begin{array}{c} {\tilde{A}}_{i}=\text{diag}\left[{\tilde{\sigma }}_{i11},\cdots ,{\tilde{\sigma }}_{1n_{i}n_{i}}\right]=\text{diag}{\left[\tau \left(1-\tau \right),\cdots ,\tau \left(1-\tau \right)\right]}_{n_{i}\times n_{i}} \end{array}$$ (12)

The general stationary auto-correlation matrix, *Σ*_*i*_(*ρ*), accommodates the correlations of many stationary dynamics such as auto-regressive order 1 (AR(1)), moving average order 1 (MA(1)), and equi-correlations (EQC) models.

Finding the solution for (11) is very difficult because the objective function *Ψ*(*β*_*τ*_, *U*) is neither convex nor continuous and could not be differentiated. Although several methods do not require differentiable and continuous estimating functions, they are very complicated and computationally demanding. To avoid these difficulties, we apply the induced smoothing method [15], which leads to the following continuous estimating equation

$$\begin{array}{c} \tilde{\Psi }\left(\beta_{\tau },U\right)=\sum_{i=1}^{m}X_{i}^{T}\Delta_{i}\Gamma_{i}\Sigma_{i}^{-1}\left(\rho \right){\tilde{\psi }}_{\tau }\left(\epsilon_{i}\right), \end{array}$$ (13)

where

$$\begin{array}{c} {\tilde{\psi }}_{\tau }\left(\epsilon_{i}\right)={\left(\tau -1+\Phi \left(\frac{b_{i1}}{r_{i1}}\right),\cdots ,\tau -1+\Phi \left(\frac{b_{in_{i}}}{r_{in_{i}}}\right)\right)}^{T}, \end{array}$$

with $b_{ij}=\log\left(y_{ij}/\left(U_{ij}\mu_{ij}^{\tau }\right)\right)$, $r_{ij}=\sqrt{x_{ij}^{T}\Omega x_{ij}}$ for *j* = 1, …, *n*_*i*_, *Ω* being an estimate of covariance matrix of *β*_*τ*_ and *Φ*(⋅) being the CDF of standard normal distribution.

To use the BLUPs of random effects, ${\hat{U}}_{*}$ and ${\hat{U}}_{**}$, updated by (4) and (5), a Taylor expansion of (13) at $U=\hat{U}$ gives

$$\begin{array}{c} \tilde{\Psi }\left(\beta_{\tau },U\right)=\tilde{\Psi }\left(\beta_{\tau },\hat{U}\right)+\frac{\partial \tilde{\Psi }\left(\beta_{\tau },U\right)}{\partial U}{|}_{U=\hat{U}}\left(U-\hat{U}\right), \end{array}$$ (14)

where

$$\begin{array}{c} \tilde{\Psi }\left(\beta_{\tau },\hat{U}\right)=\sum_{i=1}^{m}X_{i}^{T}{\hat{\Delta }}_{i}\Gamma_{i}\Sigma_{i}^{-1}\left(\rho \right){\tilde{\psi }}_{\tau }\left(\hat{\epsilon_{i}}\right), \end{array}$$ (15)

and ${\hat{\Delta }}_{i}$ and $\hat{\varepsilon_{i}}$ are obtained from Δ_*i*_ and *ϵ*_*i*_ with *U*_*ij*_ replaced by ${\hat{U}}_{ij}$, for *j* = 1, …, *n*_*i*_. The first order derivative of (13) with respect to *U* at $\hat{U}$ is

$$\begin{array}{c} \frac{\partial \tilde{\Psi }\left(\beta_{\tau },U\right)}{\partial U}{|}_{U=\hat{U}}=-\sum_{i=1}^{m}X_{i}^{T}O_{i}\Gamma_{i}\Sigma_{i}^{-1}\left(\rho \right){\tilde{\Lambda }}_{i}, \end{array}$$

where $O_{i}=\text{diag}\left[\mu_{i1}^{\tau },\ldots ,\mu_{in_{i}}^{\tau }\right]$, ${\tilde{\Lambda }}_{i}=\text{diag}\left[\phi \left(b_{i1}/r_{i1}\right)/r_{i1},\ldots ,\phi \left(b_{in_{i}}/r_{in_{i}}\right)/r_{in_{i}}\right]{|}_{U_{ij}={\hat{U}}_{ij}}$ and *ϕ*(⋅) being the pdf of standard normal distribution.

Since $\hat{U}$ is unbiased, $E(U-\hat{U})=0$, quantile estimating equation can be approximated by (15). Moreover, we have $\left(U_{i}-{\hat{U}}_{i}\right)=E{\left(U_{i}-{\hat{U}}_{i}\right)}^{2}=c_{i}$ and $\left(U_{ij}-{\hat{U}}_{ij}\right)=E{\left(U_{ij}-{\hat{U}}_{ij}\right)}^{2}=c_{ij}$, and when only one level of random effects is considered, $\text{cov}\left(U_{ij}-{\hat{U}}_{ij},U_{is}-{\hat{U}}_{is}\right)=\text{cov}\left(U_{i}-{\hat{U}}_{i},U_{i}-{\hat{U}}_{i}\right)=c_{i}$ for *j*, *s* = 1, …, *n*_*i*_.

Furthermore, the first order derivative of the estimating objectives in (14) with respect to *β*_*τ*_ is

$$\begin{array}{c} \frac{\partial \tilde{\Psi }\left(\beta_{\tau },U\right)}{\partial \beta_{\tau }}=-\sum_{i=1}^{m}X_{i}^{T}{\hat{\Delta }}_{i}\Gamma_{i}\Sigma_{i}^{-1}\left(\rho \right){\tilde{\Lambda }}_{i}X_{i}. \end{array}$$ (16)

By using the Newton-Raphson algorithm, the smoothed estimators of *β*_*τ*_ and the conditional covariance matrix *Ω* can be updated by

$$\begin{array}{c} {\tilde{\beta }}_{\tau }^{*}={\tilde{\beta }}_{\tau }+{\left[-\frac{\partial \tilde{\Psi }\left(\beta_{\tau },U\right)}{\partial \beta_{\tau }}\right]}^{-1}\times [\tilde{\Psi }\left(\beta_{\tau },\hat{U}\right)],\;\text{and} \end{array}$$

$$\begin{array}{c} {\tilde{\Omega }}^{*}={\left[-\frac{\partial \tilde{\Psi }\left(\beta_{\tau },U\right)}{\partial \beta_{\tau }}\right]}^{-1}\times [\text{cov}(\tilde{\Psi }\left(\beta_{\tau },U\right))]\times {\left[-\frac{\partial \tilde{\Psi }\left(\beta_{\tau },U\right)}{\partial \beta_{\tau }}\right]}^{-1}, \end{array}$$

where

$$\begin{array}{c} \begin{array}{cc} \text{cov}(\tilde{\Psi }\left(\beta_{\tau },U\right)) & =\sum_{i=1}^{m}X_{i}^{T}{\hat{\Delta }}_{i}\Gamma_{i}\Sigma_{i}^{-1}\left(\rho \right){\tilde{\psi }}_{\tau }\left(\hat{\epsilon_{i}}\right){\tilde{\psi }}_{\tau }^{T}\left(\hat{\epsilon_{i}}\right)\Sigma_{i}^{-1}\left(\rho \right)\Gamma_{i}{\hat{\Delta }}_{i}X_{i}\\ & +\sum_{i=1}^{m}X_{i}^{T}O_{i}\Gamma_{i}\Sigma_{i}^{-1}\left(\rho \right){\tilde{\Lambda }}_{i}C_{i}{\tilde{\Lambda }}_{i}\Sigma_{i}^{-1}\left(\rho \right)\Gamma_{i}O_{i}X_{i} \end{array} \end{array}$$

and $C_{i}=c_{i}\cdot I_{n_{i}\times n_{i}}$ if *ν*^2^ = 0, $C_{i}=\text{diag}\left[c_{i1},\ldots ,c_{in_{i}}\right]$ if *ν*^2^ ≠ 0, until convergence. The unconditional standard deviations of *β*_*τ*_ can be estimated by bootstrap method.

## Asymptotic properties

In this section, we derive asymptotic distributions of the proposed estimates ${\hat{\beta }}_{\tau }$ and ${\tilde{\beta }}_{\tau }$ obtained from the estimating functions (11) and (13) respectively.

**Theorem 11**. *Under regularity conditions A1-A5 listed in the* S1 Appendix, *the estimator* ${\hat{\beta }}_{\tau }$ is $\sqrt{m}$ *-consistent and asymptotically normal, i.e*.

$$\begin{array}{c} \sqrt{m}\left({\hat{\beta }}_{\tau }-\beta_{\tau }\right)\to N\left(0,G^{-1}\left(\beta_{\tau }\right)V{\left\{G^{-1}\left(\beta_{\tau }\right)\right\}}^{T}\right), \end{array}$$

where

$$\begin{array}{cc} G(\beta_{\tau })= & \lim_{m\to \infty }\frac{1}{m}\sum_{i=1}^{m}X_{i}^{T}\Delta_{i}\Gamma_{i}\Sigma_{i}^{-1}\left(\rho \right)\Gamma_{i}\Delta_{i}X_{i}, \end{array}$$

$$\begin{array}{cc} V= & \lim_{m\to \infty }\frac{1}{m}\sum_{i=1}^{m}X_{i}^{T}\Delta_{i}\Gamma_{i}\Sigma_{i}^{-1}\left(\rho \right)\text{cov}\left\{\psi_{\tau }\left(\epsilon_{i}\right)\right\}\Sigma_{i}^{-1}\left(\rho \right)\Gamma_{i}\Delta_{i}X_{i}. \end{array}$$

*Under regularity conditions A1-A5 listed in the S1 Appendix, the smoothed estimating function* $\tilde{\Psi }\left(\beta_{\tau },U\right)$ *is equivalent to the estimating function Ψ*(*β*_*τ*_, *U*), *i.e*.

$$\begin{array}{c} \frac{1}{\sqrt{m}}\left\{\tilde{\Psi }\left(\beta_{\tau },U\right)-\Psi \left(\beta_{\tau },U\right)\right\}=o_{p}\left(1\right). \end{array}$$

**Theorem 2**. *Under regularity conditions A1-A5 listed in the* S1 Appendix, *the estimator* ${\tilde{\beta }}_{\tau }$ is $\sqrt{m}$ *-consistent and asymptotically normal, i.e*.

$$\begin{array}{c} \sqrt{m}\left({\tilde{\beta }}_{\tau }-\beta_{\tau }\right)\to N\left(0,G^{-1}\left(\beta_{\tau }\right)V{\left\{G^{-1}\left(\beta_{\tau }\right)\right\}}^{T}\right), \end{array}$$ (38)

*where G*(*β*_*τ*_) *and V are defined as in Theorem 1*.

Proofs are deferred to the S1 Appendix.

Theorem 1 and Theorem 2 imply the asymptotic equivalence of the estimators obtained from the smoothed and original estimating equations.

## A real data analysis

In this section we illustrate our proposed quantile mixed regression model by analyzing the panel data with two-week seizure counts for 59 epileptics presented by [1]. This data is classic and available in the R package *MASS*, and can be applied on to compare our results with others’ in the literature. The number of seizures was recorded for a baseline period of 8 weeks, and then patients were randomly assigned to an anti-epileptic drug treatment group (*Trt*=1) or a control group (*Trt*=0). Further counts of seizures were then taken during the two-week periods before four successive visits to the clinic. Preliminary analysis suggests much lower counts during the fourth visit than other visits, we use a linear trend covariate Visit (coded (-0.3,-0.1,0.1,0.3)) introduced by [21].

Let *y*_*ij*_ be the seizure count for patient *i* on the *j*th visit, *Base* be the logarithm of a quarter of baseline seizure counts, *Trt* be the treatment indicator, *Age* be the logarithm of age, *Visit* be the visit time. We take the *Base*, *Trt*, *Age*, *Visit*, and the interaction term *Base*.*Trt* as the covariates, while *u*_*i*_ and *u*_*ij*_ are random effects at patient and visit levels respectively. Our analysis is based on a Poisson mixed quantile regression model using BLUPs of random effects as

$$\begin{array}{c} Y_{ij}|U=u∼Poisson\left(u_{ij}\mu_{ij}\right) \end{array}$$

and

$$\begin{array}{c} Q_{\tau }\left(Y_{ij}|U=u\right)=u_{ij}\mu_{ij}^{\tau }, \end{array}$$

where *μ*_*ij*_ = exp(*β*_0_ + *β*_1_×*Base* + *β*_2_×*Trt* + *β*_3_×*Age* + *β*_4_×*Visit* + *β*_5_×*Base*.*Trt*), $\mu_{ij}^{\tau }=\exp\left(\beta_{0\tau }+\beta_{1\tau }\times Base+\beta_{2\tau }\times Trt+\beta_{3\tau }\times Age+\beta_{4\tau }\times Visit+\beta_{5\tau }\times Base.Trt\right)$; *β* and *β*_*τ*_ are regression parameters.

We fit our proposed quantile regression model for three quantiles, *τ* = 0.25, 0.50 and 0.85, respectively, and for two different dispersion structures, *ν*^2^ = 0 and *ν*^2^ ≠ 0. We report the estimated parameters (*EP*), their asymptotic standard errors (*SE*) and their 95% confidence intervals (*CI*) in Table 1. For both dispersion structures *ν*^2^ = 0 and *ν*^2^ ≠ 0, the results of the mean regression parameter and dispersion parameter estimates are comparable with those from previous studies. [1, 18] As indicated, the predicted mean counts of seizure for the treatment group can be either higher or lower than the counts in the control group depending on whether the baseline exceeds a threshold or not. [18] The anti-epileptic drug may be restricted to patients with high baseline counts.

**Table 1:** Estimated regression and dispersion parameters (EP), their standard errors (SE) and corresponding 95% confidence intervals (CI) from fitting the proposed Poisson mixed quantile regression model for two different dispersion parameter structures, *ν*^2^ = 0 and *ν*^2^ ≠ 0, with *τ* = 0.25, 0.5, 0.85.

*τ*	Parameter	*ν*^2^ = 0			*ν*^2^ ≠ 0		
		EP	SE	CI	EP	SE	CI
	*β*_0_	-1.59	1.24	(-4.02, 0.85)	-1.23	1.21	(-3.61, 1.15)
	*β*_1_	0.90	0.14	(0.63, 1.17)	0.87	0.14	(0.60, 1.14)
	*β*_2_	-0.90	0.45	(-1.78, -0.03)	-0.84	0.41	(-1.64, -0.04)
	*β*_3_	0.57	0.36	(-0.14, 1.28)	0.48	0.36	(-0.21, 1.18)
	*β*_4_	-0.30	0.17	(-0.63, 0.03)	-0.26	0.19	(-0.63, 0.10)
	*β*_5_	0.35	0.22	(-0.07, 0.78)	0.32	0.21	(-0.09, 0.74)
	*σ*^2^		0.21			0.22	
	*ν*^2^				0.28		
0.25	*β*_0*τ*_	-2.82	1.59	(-5.95, 0.30)	-0.66	1.09	(-2.80, 1.47)
	*β*_1*τ*_	1.01	0.19	(0.66, 1.36)	0.79	0.12	(0.55, 1.03)
	*β*_2*τ*_	-1.12	0.67	(-2.43, 0.19)	-0.75	0.46	(-1.65, 0.16)
	*β*_3*τ*_	0.74	0.42	(-0.08, 1.57)	0.40	0.31	(-0.20, 1.01)
	*β*_4*τ*_	-0.21	0.23	(-0.65, 0.24)	-0.28	0.18	(-0.62, 0.07)
	*β*_5*τ*_	0.46	0.27	(-0.07, 0.98)	0.28	0.24	(-0.19, 0.75)
0.5	*β*_0*τ*_	-1.56	1.00	(-3.52, 0.39)	-1.19	0.92	(-3.00, 0.61)
	*β*_1*τ*_	0.89	0.13	(0.65, 1.15)	0.86	0.11	(0.64, 1.08)
	*β*_2*τ*_	-0.88	0.49	(-1.83, 0.08)	-0.83	0.37	(-1.56, -0.11)
	*β*_3*τ*_	0.55	0.30	(-0.03, 1.13)	0.47	0.28	(-0.07, 1.01)
	*β*_4*τ*_	-0.28	0.21	(-0.70, 0.14)	-0.26	0.26	(-0.57, 0.05)
	*β*_5*τ*_	0.34	0.25	(-0.14, 0.83)	0.32	0.20	(-0.07, 0.71)
0.85	*β*_0*τ*_	-0.96	1.11	(-3.13, 1.22)	-0.86	0.86	(-2.55, 0.83)
	*β*_1*τ*_	0.82	0.18	(0.48, 1.17)	0.83	0.13	(0.57, 1.09)
	*β*_2*τ*_	-0.84	0.54	(-1.91, 0.22)	-0.82	0.41	(-1.62, -0.01)
	*β*_3*τ*_	0.54	0.33	(-0.11, 1.18)	0.43	0.26	(-0.08, 0.94)
	*β*_4*τ*_	-0.54	0.18	(-0.89, -0.18)	-0.28	0.18	(-0.62, 0.07)
	*β*_5*τ*_	0.31	0.27	(-0.21, 0.83)	0.32	0.21	(-0.08, 0.72)

Comparing to other alternative methods using mean regression models, our proposed model allows us to examine the effects of a factor at different levels of the response distribution. By taking different quantiles of the seizure count response variable, we tend to capture more hidden features of the covariate effects on the seizure count. At the 0.25th quantile, the counts of seizures depends more on the baseline for both models with and without visit-specific random effects. The drug treatment does not show significant effects. At higher quantiles (*τ*=0.50, 0.85), when we consider the random effects at visit levels (*ν*^2^ ≠ 0), the anti-epileptic drug tend to have significant effect to control seizure counts. By introducing the visit-level random effects *U*_*ij*_, we are able to capture the dependence of observations between visits or the trajectory of measurements over time for each patient. Therefore, the effect of the drug could be estimated more accurately to indicate whether there is a drop of seizure counts over time.

Accepting significant level slightly larger than 0.05, visit time also have significant effects on seizure counts if we take the visit-level random effects in the model. The differences of the estimates of model parameters between the models with and without visit-specific random effects, especially the intercept terms are remarkable. Our simulation results in the following section reveals that ignoring visit-specific random effects is the main reason of it

## Simulation studies

In this section, we conduct a simulation study mimicking the seizure count data to examine the performance of our proposed approach and compare the simulation results with other methods in the literature. We take *Base*, *Trt*, *Age*, *Visit* and the interaction *Base*.*Trt* as our covariates. We generate a dataset of 10000 subjects, and their seizure count response variables following a Poisson mixed model. We specify the parameter values of *β*_0_ = −1.30, *β*_1_ = 0.88, *β*_2_ = −0.88, *β*_3_ = 0.50, *β*_4_ = −0.23, *β*_5_ = 0.34, *σ*^2^ = 0.24 and *ν*^2^ = 0.44 as the true model parameters of the conditional mean regression model. The data with 10000 subjects is treated as a pseudo-population. For 500 simulation runs, we sample 59 subjects each time from this pseudo-population.

The simulated data are analysed for both dispersion structures, *ν*^2^ = 0 and *ν*^2^ ≠ 0 each at three quantiles (*τ* = 0.25, 0.50, 0.85). We report the average bias (Bias, compared to the estimated parameters of 10000 datasets), the average of estimated standard errors (ESD, using bootstrap for each simulation) of the parameters over 500 simulations in Table 2. We calculated coverage percentages of 95% confidence intervals for regression parameters using asymptotic normality of their estimators. The coverage percentages of 95% prediction intervals for random effects (*u*_1_ and *u*_11_) are also reported. Estimated coverages of 95% intervals denoted as *CP*_0.95_ are reported in Table 2. To compare our method with other methods, we also report the results for fitting a generalized linear mixed-effects model (GLMM) using **glmer** function in the R package **lme4** and fitting a linear quantile mixed model (LQMM) using the R-package **lqmm**. [22, 23] Both GLMM and LQMM are random intercept models, where the GLMM is with a logarithm link function and the LQMM utilizes the logarithm of seizure counts (excluding zero values) as response. The results of estimates for fixed-effect coefficients and the variance of the random effects, *σ*^2^, are reported in Table 3.

**Table 2:** Average bias (Bias), average of estimated standard errors (ESD) of the parameters and coverage percentages of 95% intervals (*CP*_0.95_) over 500 simulations at quantiles 0.25, 0.5, 0.85.

*τ*	Parameter	*ν*^2^ = 0			*ν*^2^ ≠ 0		
		Bias	ESD	*CP*_0.95_	Bias	ESD	*CP*_0.95_
	*β*_0_	0.04	1.07	0.95	0.00	1.25	0.95
	*β*_1_	-0.01	0.12	0.94	-0.03	0.14	0.94
	*β*_2_	0.02	0.37	0.95	0.00	0.42	0.94
	*β*_3_	-0.01	0.31	0.95	0.01	0.37	0.95
	*β*_4_	0.00	0.10	0.89	0.02	0.23	0.95
	*β*_5_	-0.02	0.18	0.95	0.01	0.22	0.93
	*U*_1_			0.97			0.98
	*U*_11_						0.95
	*σ*^2^	-0.03			-0.04		
	*ν*^2^				-0.01		
0.25	*β*_0*τ*_	-0.24	3.14	0.98	0.09	1.19	0.95
	*β*_1*τ*_	0.01	0.45	0.98	-0.02	0.17	0.96
	*β*_2*τ*_	-0.17	1.88	0.99	0.06	0.51	0.95
	*β*_3*τ*_	0.03	0.82	0.98	-0.02	0.33	0.94
	*β*_4*τ*_	-0.05	0.72	0.97	0.01	0.22	0.94
	*β*_5*τ*_	0.06	0.73	0.99	-0.02	0.24	0.96
0.50	*β*_0*τ*_	-0.11	1.48	0.95	0.05	1.18	0.93
	*β*_1*τ*_	-0.01	0.20	0.97	-0.02	0.16	0.94
	*β*_2*τ*_	-0.06	0.68	0.97	-0.02	0.50	0.94
	*β*_3*τ*_	0.02	0.42	0.95	-0.01	0.34	0.93
	*β*_4*τ*_	0.01	0.34	0.94	0.01	0.22	0.95
	*β*_5*τ*_	0.02	0.30	0.96	0.02	0.24	0.94
0.85	*β*_0*τ*_	0.23	1.21	0.95	-0.02	1.14	0.94
	*β*_1*τ*_	-0.04	0.16	0.94	-0.02	0.15	0.95
	*β*_2*τ*_	-0.02	0.54	0.98	-0.04	0.49	0.95
	*β*_3*τ*_	-0.02	0.34	0.95	0.02	0.33	0.95
	*β*_4*τ*_	0.00	0.30	0.95	-0.01	0.21	0.96
	*β*_5*τ*_	0.02	0.25	0.97	0.02	0.23	0.95

**Table 3:** Average bias (Bias), average of estimated standard errors (ESD) of the parameters and coverage percentages of 95% intervals (*CP*_0.95_) over 500 simulations for GLMM and LQMM models.

Model	Parameter	Bias	ESD	*CP*_0.95_
GLMM	*β*_0_	-0.25	1.24	1.00
	*β*_1_	0.02	0.16	0.93
	*β*_2_	-0.04	0.50	0.96
	*β*_3_	0.01	0.36	1.00
	*β*_4_	-0.07	0.09	0.34
	*β*_5_	0.00	0.24	0.96
	*σ*^2^	0.20		
LQMM	*β*_0*τ*_	-0.11	1.17	0.96
*τ* = 0.25	*β*_1*τ*_	-0.09	0.17	0.95
	*β*_2*τ*_	0.22	0.43	1.00
	*β*_3*τ*_	-0.18	0.34	0.96
	*β*_4*τ*_	-0.09	0.24	0.96
	*β*_5*τ*_	-0.12	0.23	1.00
	*σ*^2^	-0.11		
LQMM	*β*_0*τ*_	0.09	1.17	1.00
*τ* = 0.50	*β*_1*τ*_	-0.05	0.17	0.96
	*β*_2*τ*_	0.23	0.44	1.00
	*β*_3*τ*_	-0.08	0.34	0.92
	*β*_4*τ*_	-0.08	0.24	0.89
	*β*_5*τ*_	-0.10	0.22	1.00
	*σ*^2^	-0.09		
LQMM	*β*_0*τ*_	0.06	1.16	0.96
*τ* = 0.85	*β*_1*τ*_	0.05	0.15	0.96
	*β*_2*τ*_	0.23	0.43	1.00
	*β*_3*τ*_	0.10	0.33	0.96
	*β*_4*τ*_	-0.10	0.23	0.92
	*β*_5*τ*_	-0.12	0.21	1.00
	*σ*^2^	-0.20		

As in Table 2, the results of mean regression parameters are in accordance with those from [18], except that the dispersion parameters *σ*^2^ and *ν*^2^ are both just slightly underestimated. Compared to GLMM models in Table 3, our proposed method gives much smaller biases for the estimates of *β*_0_ and the variance of random effects, *σ*^2^. Moreover, we obtain better coverage probabilities which are all around 0.95 for fixed-effects parameters.

From the results of the simulation we can observe that, ignoring the visit-specific random effects, or say set *ν*^2^ = 0, leads to high estimation bias of the intercept coefficient, as well as its standard deviation of the estimator. The standard deviations of the estimators of other parameters are inflated, too. This leads to higher coverage percentages of the 95% confidence intervals than their nominal level. When both subject-specific and visit-specific random effects are considered, all estimates of parameters have small biases and smaller standard errors compared to the model with only one level of random effects. This agrees with the findings in the real data analysis in the previous section. Compared to results in Table 3, our model, especially with *ν*^2^ ≠ 0, outperforms the LQMM with smaller biases in estimating the fixed-effect coefficients and the variance of random effects, *σ*^2^. Our model is also good in estimating the standard errors of fixed-effect coefficients, ESD, and hence leads better 95% confidence interval coverage probabilities.

**Fig 1:**
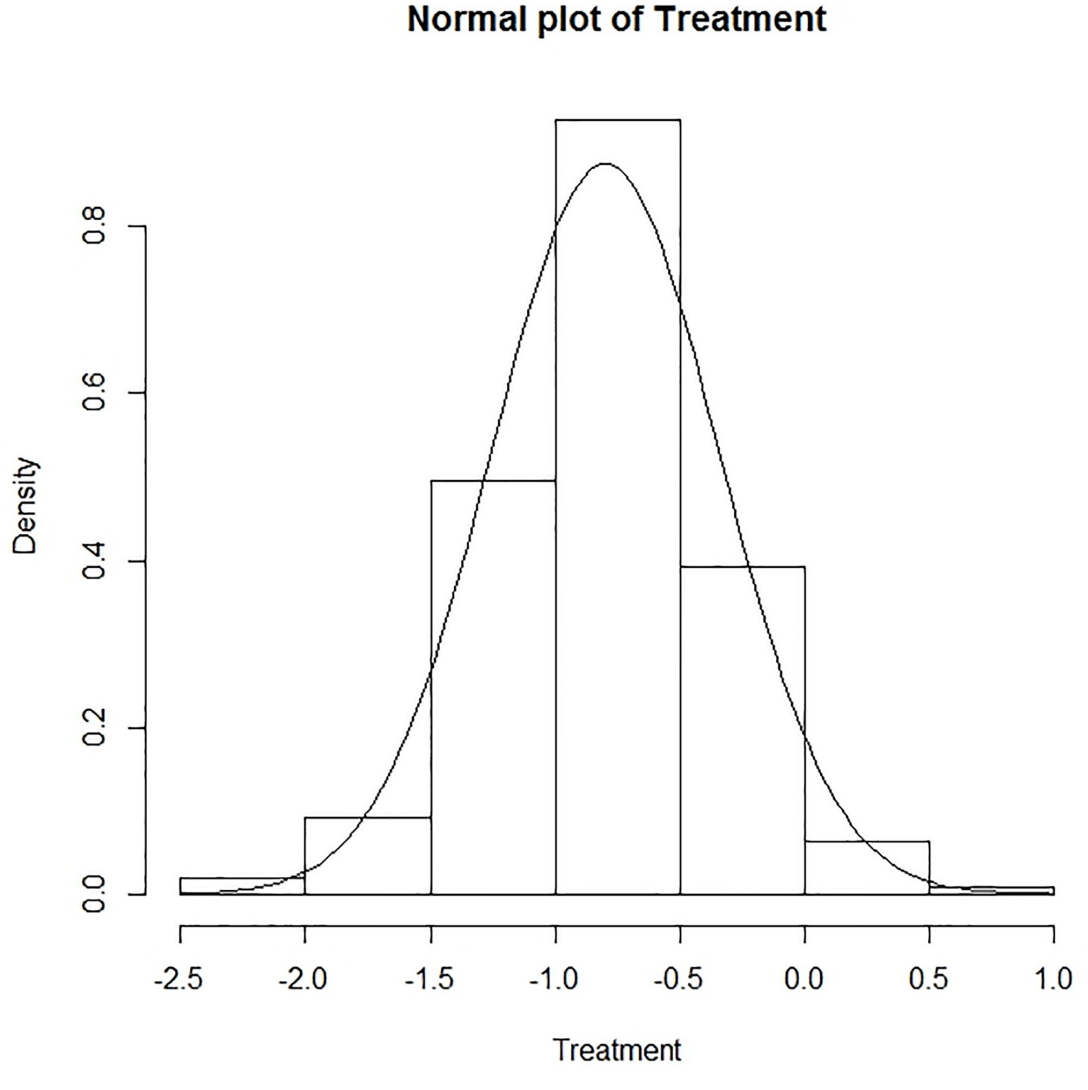
Normal plot of treatment. Normal plot of estimates for *β*_2_ under the model *ν*^2^ ≠ 0.

As we can see in Table 2, the estimated coverage percentages of 95% confidence intervals are around 0.95 except for a few parameters with values of as high as 0.99 at *τ* = 0.25 for the model without consideration of visit-level random effects with *ν*^2^ = 0. This indicates that our estimators of the regression parameters and the estimates of their standard deviations work well. Furthermore, from the normal plots of the estimates of the regression parameters (Fig 1 shows a normal plot of estimates for *β*_2_ under the model *ν*^2^ ≠ 0), our proposed estimators are approximately normally distributed and inferences based on it are reliable.

## Conclusion

In this paper, we have proposed a generalized linear mixed quantile regression model for panel data. By assuming Tweedie exponential dispersion distributions, we predict the subject-specific and visit-specific random effects by their orthodox BLUPs and treat them as fixed values in the quantile regression parameter estimation process. In order to account for the correlations between repeated measures, we applied a general stationary auto-correlation structure to the estimating equations. To reduce computational burden caused by the non-continuous estimating functions, along with Newton-Raphson iteration technique, we have extended the induced smoothing method to quantile regression. Our simulation studies reveal that our proposed method performs well. As an illustration, the proposed quantile regression estimator is applied to a real data set.

In this paper, we take both subject-specific and visit-specific random effects into consideration in quantile regression modelling. We obtain BLUPs of random effects and mean regression parameters prior to the estimation of quantile regression parameters. The prediction of random effects are not updated in the quantile estimation process. Therefore, we may develop our proposed quantile mixed model to connect the prediction of random effects and the estimation of quantile regression parameters. Our method can be easily extended to accommodate quantile mixed models with response following other distributions by specifying the value of the shape parameter *q* for assumed Tweedie distributions and a proposed model for the conditional response mean (e.g. $Y_{ij}|U=u∼Tw_{0}\left(u_{ij}\mu_{ij},\gamma^{2}u_{ij}^{1-0}\right)$ which is a normal distribution with a possible $\mu_{ij}=x_{ij}^{T}\beta$).

## Supporting information

S1 Appendix Appendix. Proofs of the asymptotic theorems.
(PDF)

S1 Data (CSV)
