# Supplementary material for: Generalized linear mixed quantile regression with panel data
Source: PLoS One. 2020 Aug 11;15(8):e0237326. doi: 10.1371/journal.pone.0237326 (PMC7419003; doi:10.1371/journal.pone.0237326)
Supplement: S1 Appendix — Proofs of the asymptotic theorems. (PDF) [file pone.0237326.s001.pdf]

Appendix

In the prediction of random effects, initial values for the estimates of regression parameters are taken from standard generalized linear models, and we take initial values for random effects and dispersion parameters as follows

$$\begin{aligned}
\hat{U}_i(0) &= \frac{\frac{1}{n_i}\sum_{j=1}^{n_i} y_{ij}}{\frac{1}{m}\sum_{i=1}^{m}\frac{1}{n_i}\sum_{j=1}^{n_i} y_{ij}},\\
\hat{U}_{ij}(0) &= \frac{y_{ij}}{\frac{1}{m}\sum_{i=1}^{m}\frac{1}{n_i}\sum_{j=1}^{n_i} y_{ij}},\\
\hat{\sigma}^2(0) &= \frac{1}{m}\sum_{i=1}^{m}(\hat{U}_i - 1)^2\\
\hat{\nu}^2(0) &= \frac{1}{m}\sum_{i=1}^{m}\frac{1}{n_i}\sum_{j=1}^{n_i}(\hat{U}_{ij} - \hat{U}_i)^2\\
\hat{\gamma}^2(0) &= \frac{1}{m}\sum_{i=1}^{m}\frac{1}{n_i}\sum_{j=1}^{n_i}\frac{(y_{ij} - \hat{U}_{ij}\mu_{ij})^2}{{\mu_{ij}}^q}.
\end{aligned}$$

The following gives a set of regularity conditions used in the theorems as well as the proofs of the theorems in our paper.

A1. For every i, the number of repeated responses n_i is bounded and the dimension p of covariates x_{ij} is fixed. We treat random effects as fixed at this point. The CDFs of $F_{ij}(z) = P(\varepsilon_{ij} \le z|x_{ij})$ are absolutely continuous with continuous densities f_{ij}, and the first derivative of f_{ij} is uniformly bounded away from 0 and ∞ at point 0, $i = 1, \dots, m$; $j = 1, \dots, n_i$.

A2. The true value β_τ is an interior point within a bounded convex region $\mathfrak{B}$.

A3. Every x_i satisfies

- With any positive definite matrix W_i, $\frac{1}{m}\sum_{i=1}^{m} X_i^T \mathit{\Gamma}_i W_i \mathit{\Gamma}_i X_i$ converges to a positive definite matrix; where $\mathit{\Gamma}_i$ is an $n_i \times n_i$ diagonal matrix with elements $f_{ij}(0)$.
- $\sup_i \|x_i\| < +\infty$, with $\|\cdot\|$ denoting the Euclidean norm.

A4. Matrix $\mathit{\Omega}$ is positive definite and $\mathit{\Omega} = O(\frac{1}{m})$.

A5. The differentiation of negative $\tilde{\mathit{\Psi}}(\beta_\tau, U)$, $-\partial\tilde{\mathit{\Psi}}(\beta_\tau, U)/\partial\beta_\tau$ is positive definite with probability 1.

Proof of Theorem 1. Let $H_i^T = X_i^T \mathit{\Delta}_i \mathit{\Gamma}_i \Sigma_i^{-1}(\rho)$ and $\psi_i = \psi_\tau(\varepsilon_i)$, and the non-smooth estimating function be $\mathit{\Theta}(\hat{\beta}_\tau) = \sum_{i=1}^{m} H_i^T \psi_i$. Let $\bar{\mathit{\Theta}}(\hat{\beta}_\tau) = \sum_{i=1}^{m} H_i^T \varphi_i$, where $\varphi_i = (\tau - P(\hat{\varepsilon}_{i1} \le 0), \dots, \tau - P(\hat{\varepsilon}_{in_i} \le 0))^T$. We can obtain

$$\begin{aligned}
\frac{1}{m}(\mathit{\Theta}(\hat{\beta}_\tau) - \bar{\mathit{\Theta}}(\hat{\beta}_\tau)) &= \frac{1}{m}\sum_{i=1}^{m} H_i^T(\psi_i - \varphi_i)\\
&= \frac{1}{m}\sum_{i=1}^{m} H_i^T \begin{pmatrix} P(\hat{\varepsilon}_{i1} \le 0) - I(\hat{\varepsilon}_{i1} \le 0)\\ \vdots \\ P(\hat{\varepsilon}_{in_i} \le 0) - I(\hat{\varepsilon}_{in_i} \le 0)\end{pmatrix}\\
&= \frac{1}{m}\sum_{i=1}^{m}\sum_{j=1}^{n_i} h_{ij}[P(\hat{\varepsilon}_{ij} \le 0) - I(\hat{\varepsilon}_{ij} \le 0)],
\end{aligned}$$

where h_{ij} is a $p \times 1$ vector and $(h_{i1}, \ldots, h_{in_i}) = H_i^T$. Applying the uniform strong law of large numbers, under condition A3

$$\sup_{\hat{\beta}_\tau \in \mathfrak{B}} \left| \frac{1}{m} \sum_{i=1}^{m} \sum_{j=1}^{n_i} h_{ij}[P(\hat{\varepsilon}_{ij} \le 0) - I(\hat{\varepsilon}_{ij} \le 0)] \right| = o(m^{-1/2}) \quad \text{a.s..}$$

Therefore,

$$\sup_{\hat{\beta}_\tau \in \mathfrak{B}} \| \frac{1}{m}(\Theta(\hat{\beta}_\tau) - \bar{\Theta}(\hat{\beta}_\tau)) \| = o(m^{-1/2}) \quad \text{a.s..}$$

Moreover,

$$G_m(\beta_\tau) = -\frac{1}{m} \frac{\partial \bar{\Theta}(\hat{\beta}_\tau)}{\partial \hat{\beta}_\tau} \bigg|_{\hat{\beta}_\tau = \beta_\tau} = \frac{1}{m} \sum_{i=1}^{m} H_i^T \Gamma_i \Delta_i X_i$$

is positive definite. We have $G_m(\beta_\tau) \to G(\beta_\tau)$, with probability 1, when $m \to +\infty$. Because $P(\varepsilon_{ij} \le 0) = \tau$, β_τ is the unique solution of $\bar{\Theta}(\hat{\beta}_\tau) = 0$, together with $\Theta(\hat{\beta}_\tau) = 0$ and condition A3, it implies that $\hat{\beta}_\tau \to \beta_\tau$ as $m \to \infty$.

Since ψ_i are independent random variables with expectation zero, and $\text{var}\{\Theta(\beta_\tau)/m\} = \frac{1}{m} \sum_{i=1}^{m} X_i^T \Delta_i \Gamma_i \Sigma_i^{-1}(\rho)\, \text{cov}(\psi_i) \Sigma_i^{-1}(\rho) \Gamma_i \Delta_i X_i$, the multivariate central limit theorem implies that $\frac{1}{\sqrt{m}} \Theta(\beta_\tau) \to N(0, V)$.

For any $\hat{\beta}_\tau$ satisfying $\|\hat{\beta}_\tau - \beta_\tau\| < cm^{-1/3}$,

$$\begin{aligned} \Theta(\hat{\beta}_\tau) - \Theta(\beta_\tau) &= \sum_{i=1}^{m} H_i^T(\hat{\beta}_\tau)\psi_i(\hat{\beta}_\tau) - \sum_{i=1}^{m} H_i^T(\beta_\tau)\psi_i(\beta_\tau) \\ &= \sum_{i=1}^{m} H_i^T(\hat{\beta}_\tau)\{\psi_i(\hat{\beta}_\tau) - \psi_i(\beta_\tau)\} + \sum_{i=1}^{m} \{H_i^T(\hat{\beta}_\tau) - H_i^T(\beta_\tau)\}^T \psi_i(\beta_\tau). \end{aligned}$$

The first term can be expressed as

$$\begin{aligned} &\sum_{i=1}^{m} H_i^T(\hat{\beta}_\tau)\{\psi_i(\hat{\beta}_\tau) - \psi_i(\beta_\tau)\} \\ &\quad = \sum_{i=1}^{m} H_i^T(\hat{\beta}_\tau)\varphi_i(\hat{\beta}_\tau) + \sum_{i=1}^{m} H_i^T(\hat{\beta}_\tau)\{\psi_i(\hat{\beta}_\tau) - \psi_i(\beta_\tau) - \varphi_i(\hat{\beta}_\tau)\} \\ &\quad = \sum_{i=1}^{m} H_i^T(\hat{\beta}_\tau)\varphi_i(\hat{\beta}_\tau) + \sum_{i=1}^{m} H_i^T(\hat{\beta}_\tau)\{P(\hat{\varepsilon}_{ij} \le 0) - I(\hat{\varepsilon}_{ij} \le 0) \\ &\quad + I(\hat{\varepsilon}_{ij} \le 0) - \tau\}. \end{aligned}$$

By the Lemma in Jung (1996),

$$\sup \left| \sum_{i=1}^{m} H_i^T(\hat{\beta}_\tau)\{P(\hat{\varepsilon}_{ij} \le 0) - I(\hat{\varepsilon}_{ij} \le 0) + I(\hat{\varepsilon}_{ij} \le 0) - \tau\} \right| = o_p(\sqrt{m}).$$

Therefore,

$$\begin{aligned} \sum_{i=1}^{m} H_i^T(\hat{\beta}_\tau)\{\psi_i(\hat{\beta}_\tau) - \psi_i(\beta_\tau)\} &= \sum_{i=1}^{m} H_i^T(\hat{\beta}_\tau)\varphi_i(\hat{\beta}_\tau) + o_p(\sqrt{m}) \\ &= \bar{\Theta}(\hat{\beta}_\tau) + o_p(\sqrt{m}) \end{aligned}$$

From the law of large numbers, the second term can be expressed as

$$\begin{aligned}\sum_{i=1}^{m}\{H_i^T(\hat\beta_\tau)-H_i^T(\beta_\tau)\}^T\psi_i(\beta_\tau) &= \sum_{i=1}^{m}\sum_{j=1}^{n_i}(h_{ij}(\hat\beta_\tau)-h_{ij}(\beta_\tau))[P(\varepsilon_{ij}\le 0)-I(\varepsilon_{ij}\le 0)]\\ &= o_p(\sqrt{m}).\end{aligned}$$

Hence, $\Theta(\hat\beta_\tau)-\Theta(\beta_\tau)=\bar\Theta(\hat\beta_\tau)+o_p(\sqrt{m})$. By using Taylor's expansion of $\bar\Theta(\hat\beta_\tau)$, we have

$$\frac{1}{\sqrt{m}}\{\Theta(\hat\beta_\tau)-\Theta(\beta_\tau)\}=\frac{1}{m}\frac{\partial\bar\Theta(\hat\beta_\tau)}{\partial\hat\beta_\tau}\bigg|_{\hat\beta_\tau=\beta_\tau}\sqrt{m}(\hat\beta_\tau-\beta_\tau)+o_p(1).$$

For $\hat\beta_\tau$ being in the $m^{-1/3}$ neighbourhood of β_τ and $U(\hat\beta_\tau)=0$, it implies

$$\sqrt{m}(\hat\beta_\tau-\beta_\tau)=G_m^{-1}(\beta_\tau)\frac{1}{\sqrt{m}}U(\beta_\tau)+o_p(1).$$

Therefore $\sqrt{m}(\hat\beta_\tau-\beta_\tau)\to N(0,G^{-1}(\beta_\tau)V\{G^{-1}(\beta_\tau)\}^T)$ as $m\to+\infty$. □

Proof of Lemma 1. Let $\psi_{ij}=\psi_\tau(\varepsilon_{ij})$, $\tilde\psi_{ij}=\tilde\psi_\tau(\varepsilon_{ij})$ and $d_{ij}=b_{ij}/r_{ij}$, where $b_{ij}=\log(y_{ij}/(U_{ij}\mu_{ij}^\tau))$ and $r_{ij}=\sqrt{x_{ij}^T\Omega x_{ij}}$. Since $\tilde\psi_{ij}-\psi_{ij}=\mathrm{sgn}(-d_{ij})\Phi(-|d_{ij}|)$, where $\mathrm{sgn}(\cdot)$ is the sign function, we have

$$\begin{aligned}\frac{1}{\sqrt{m}}\{\tilde\Theta(\beta_\tau)-\Theta(\beta_\tau)\} &= \frac{1}{\sqrt{m}}\sum_{i=1}^{m}X_i^T\Delta_i\Gamma_i\Sigma_i^{-1}(\rho)\begin{pmatrix}\mathrm{sgn}(-d_{i1})\Phi(-|d_{i1}|)\\ \vdots\\ \mathrm{sgn}(-d_{in_i})\Phi(-|d_{in_i}|)\end{pmatrix}\\ &= \frac{1}{\sqrt{m}}\sum_{i=1}^{m}\sum_{j=1}^{n_i}z_{ij}\,\mathrm{sgn}(-d_{ij})\Phi(-|d_{ij}|),\end{aligned}$$

where z_{ij} is the jth column of $X_i^T\Delta_i\Gamma_i\Sigma_i^{-1}(\rho)$. Along with the facts that $\varepsilon_{ij}=y_{ij}-U_{ij}\mu_{ij}^\tau=(e^{b_{ij}}-1)U_{ij}\mu_{ij}^\tau$, $U_{ij}\mu_{ij}^\tau>0$, we have

$$\begin{aligned}E(\tilde\psi_{ij}-\psi_{ij}) &= \int_{-\infty}^{+\infty}\mathrm{sgn}(-d_{ij})\Phi(-|d_{ij}|)f_{ij}(\varepsilon)d\varepsilon\\ &= \int_{-\infty}^{+\infty}\Phi(-|b|/r_{ij})\{2I(b\le 0)-1\}f_{ij}(\varepsilon)d\varepsilon\\ &= \int_{-\infty}^{+\infty}\Phi(-|b|/r_{ij})\{2I(b\le 0)-1\}f_{ij}((e^b-1)U_{ij}\mu_{ij}^\tau)\frac{d\varepsilon}{db}db\\ &= \int_{-\infty}^{+\infty}\Phi(-|b|/r_{ij})\{2I(b\le 0)-1\}f_{ij}((e^b-1)U_{ij}\mu_{ij}^\tau)U_{ij}\mu_{ij}^\tau e^b db\\ &= \int_{-\infty}^{+\infty}\Phi(-|b|/r_{ij})\{2I(b\le 0)-1\}g_{ij}(b)db\\ &= r_{ij}\int_{-\infty}^{+\infty}\Phi(-|t|)\{2I(t\le 0)-1\}[g_{ij}(0)+g_{ij}^{'}(\zeta(t))r_{ij}t]dt,\end{aligned}$$

where $g_{ij}(b)=f_{ij}((e^b-1)U_{ij}\mu_{ij}^\tau)U_{ij}\mu_{ij}^\tau e^b$ is the density function of the random variable b, which has similar properties with density function f_{ij} (since $g_{ij}(bij)=f_{ij}(\varepsilon_{ij})y_{ij}$, $y_{ij}>0$), and $\zeta(t)$ is between 0 and $r_{ij}t$. Because $\int_{-\infty}^{+\infty}\Phi(-|t|)\{2I(t\le 0)-1\}dt=0$, we

have $r_{ij}\int_{-\infty}^{+\infty}\Phi(-|t|)\{2I(t\le 0)-1\}g_{ij}(0)dt=0$. Since $\int_{-\infty}^{+\infty}|t|\Phi(-|t|)dt=1/2$, and by condition A1, there exists a constant M such that $\sup_{ij}|g_{ij}^{'}(\zeta(t))|\le M$. Therefore,

$$\begin{aligned}|E(\tilde{\psi}_{ij}-\psi_{ij})| &\le r_{ij}^2\int_{-\infty}^{+\infty}|t|\Phi(-|t|)|g_{ij}^{'}(\zeta(t))|dt\\ &\le Mr_{ij}^2/2.\end{aligned}$$

Under regularity conditions A3 and A4, when $m\to+\infty$,

$$\left\|\frac{1}{\sqrt{m}}E\{\tilde{\Theta}(\beta_\tau)-\Theta(\beta_\tau)\}\right\|\le\frac{1}{\sqrt{m}}\sup_{i,j}|z_{ij}|\sum_{i=1}^{m}Mr_{ij}^2/2=o(1).$$

Moreover,

$$\frac{1}{m}\operatorname{var}\{\tilde{\Theta}(\beta_\tau)-\Theta(\beta_\tau)\}=\frac{1}{m}\sum_{i=1}^{m}\operatorname{var}\left\{\sum_{j=1}^{n_i}z_{ij}\operatorname{sgn}(-d_{ij})\Phi(-|d_{ij}|)\right\}.$$

According to Cauchy-Schwartz inequality,

$$\begin{aligned}\frac{1}{m}\operatorname{var}\{\tilde{\Theta}(\beta_\tau)-\Theta(\beta_\tau)\}\le&\frac{1}{m}\sum_{i=1}^{m}\sum_{j=1}^{n_i}z_{ij}z_{ij}^T\operatorname{var}(\tilde{\psi}_{ij}-\psi_{ij})\\ &+\frac{1}{m}\sum_{i=1}^{m}\sum_{j=1}^{n_i}\sum_{k\ne j}^{n_i}z_{ij}z_{ik}^T\sqrt{\operatorname{var}(\tilde{\psi}_{ij}-\psi_{ij})\operatorname{var}(\tilde{\psi}_{ik}-\psi_{ik})}.\end{aligned}$$

Hence for each $j=1,\dots,n_i$,

$$\begin{aligned}\operatorname{var}(\tilde{\psi}_{ij}-\psi_{ij})\le E(\tilde{\psi}_{ij}-\psi_{ij})^2&=\int_{-\infty}^{+\infty}\{\operatorname{sgn}(-d_{ij})\Phi(-|d_{ij}|)\}^2g_{ij}(b)db\\ &=r_{ij}\int_{-\infty}^{+\infty}\Phi^2(-|t|)g_{ij}(r_{ij}t)dt\\ &=r_{ij}\int_{|t|>\Upsilon}\Phi^2(-|t|)g_{ij}(r_{ij}t)dt+r_{ij}\int_{|t|\le\Upsilon}\Phi^2(-|t|)g_{ij}(r_{ij}t)dt\\ &\le\Phi^2(-\Upsilon)+r_{ij}\Upsilon f_{ij}(\zeta),\end{aligned}$$

where Υ is positive, and ζ is in the interval $(-r_{ij}\Upsilon,r_{ij}\Upsilon)$. Let $\Upsilon=m^{1/3}$. Under condition A4, and since $r_{ij}=O(m^{-1/2})$, it implies $r_{ij}\Upsilon=O(m^{-1/6})$. Moreover, both $\Phi^2(-\Upsilon)$ and $r_{ij}\Upsilon f_{ij}(\zeta)$ converges to 0 as $m\to+\infty$. By conditions A2 and A3, we obtain that $\frac{1}{m}\operatorname{var}\{\tilde{\Theta}(\beta_\tau)-\Theta(\beta_\tau)\}=o(1)$. Therefore, for any β_τ, we have $\frac{1}{\sqrt{m}}\{\tilde{\Theta}(\beta_\tau)-\Theta(\beta_\tau)\}\to0$ in probability as $m\to+\infty$. □

Proof of Theorem 2. Using the results in Theorem 1 along with $\sup_{\beta_\tau\in\mathfrak{B}}\|m^{-1}\{\Theta(\beta_\tau)-\bar{\Theta}(\beta_\tau)\}\|=o(m^{-1/2})$ a.s., and by the triangle inequality, we can obtain that $\sup_{\beta_\tau\in\mathfrak{B}}\|m^{-1}\{\tilde{\Theta}(\beta_\tau)-\bar{\Theta}(\beta_\tau)\}\|=o(m^{-1/2})$. Let β_τ as the unique solution of equation $\bar{\Theta}(\beta_\tau)=0$ and $\tilde{\beta}_\tau$ solves $\tilde{\Theta}(\beta_\tau)=0$, we have $\tilde{\beta}_\tau\to\beta_\tau$ as $m\to+\infty$.

In order to prove the asymptotic normality of $\tilde{\beta}_\tau$, we first prove that $m^{-1}\{\tilde{G}(\beta_\tau)-G(\beta_\tau)\}\xrightarrow{p}0$, where $\tilde{G}(\beta_\tau)=-\partial\tilde{\Theta}(\beta_\tau)/\partial\beta_\tau=\sum_{i=1}^{m}X_i^T\Delta_i\Gamma_i\Sigma_i^{-1}(\rho)\tilde{\Lambda}_iX_i$. Let $H_i^T=X_i^T\Delta_i\Gamma_i\Sigma_i^{-1}(\rho)=(h_{i1},\dots,h_{in_i})$, where h_{ij} is a $p\times1$ vector, we have

$$E\{\tilde{G}(\beta_\tau)\}-G(\beta_\tau)=\sum_{i=1}^{m}\sum_{j=1}^{n_i}h_{ij}\left\{\frac{1}{r_{ij}}E\phi\left(\frac{b_{ij}}{r_{ij}}\right)-g_{ij}(0)\right\}x_{ij}.$$

Because

$$\left|\frac{1}{r_{ij}}E\phi\left(\frac{b_{ij}}{r_{ij}}\right)-g_{ij}(0)\right| = \left|\frac{1}{r_{ij}}\int_{-\infty}^{+\infty}\phi\left(\frac{b}{r_{ij}}\right)g_{ij}(b)db-g_{ij}(0)\right|$$
$$= \left|\int_{-\infty}^{+\infty}\phi(t)\{g_{ij}(0)+r_{ij}tg_{ij}(\xi_t)\}dt-g_{ij}(0)\right|$$
$$= \left|r_{ij}\int_{-\infty}^{+\infty}\phi(t)tg_{ij}(\xi_t)dt\right|$$
$$\leq r_{ij}\int_{-\infty}^{+\infty}|\phi(t)tg_{ij}(\xi_t)|dt,$$

where ξ_t is between 0 and $r_{ij}t$. Under condition A1, there is a constant M such that $g_{ij}(\xi_t) \leq M$. Furthermore, according to condition A4, we can obtain

$$\left|\frac{1}{r_{ij}}E\phi\left(\frac{b_{ij}}{r_{ij}}\right)-g_{ij}(0)\right| \leq \sqrt{\frac{2}{\pi}}r_{ij}M \to 0.$$

By the strong law of large numbers, we have $m^{-1}\tilde{G}(\beta_\tau) \to E\{m^{-1}\tilde{G}(\beta_\tau)\}$. Using the triangle inequality, we can obtain that

$$|m^{-1}\{\tilde{G}(\beta_\tau)-G(\beta_\tau)\}| \leq |m^{-1}\{\tilde{G}(\beta_\tau)-E\tilde{G}(\beta_\tau)\}|+|m^{-1}\{E\tilde{G}(\beta_\tau)-G(\beta_\tau)\}| \to o(1),$$

which is equivalent to $m^{-1}\{\tilde{G}(\beta_\tau)-G(\beta_\tau)\} \xrightarrow{p} 0$.

By Taylor series expansion of $\tilde{U}(\hat{\beta}_\tau)$ around β_τ, we have

$$\tilde{\Theta}(\hat{\beta}_\tau) = \tilde{\Theta}(\beta_\tau) - \tilde{G}(\hat{\beta}_\tau^*)(\hat{\beta}_\tau-\beta_\tau),$$

where $\hat{\beta}_\tau^*$ lies between $\hat{\beta}_\tau$ and β_τ. Let $\hat{\beta}_\tau = \tilde{\beta}_\tau$. Because $\tilde{\Theta}(\tilde{\beta}_\tau) = 0$ and $\tilde{\beta}_\tau \to \beta_\tau$, we therefore have $\hat{\beta}_\tau^* \to \beta_\tau$ and $\tilde{G}(\hat{\beta}_\tau^*) \to \tilde{G}(\beta_\tau)$. By Lemma 1 and $m^{-1}\{\tilde{G}(\beta_\tau)-G(\beta_\tau)\} \xrightarrow{p} 0$, it thus implies

$$\sqrt{m}(\tilde{\beta}_\tau-\beta_\tau) = G_m^{-1}(\beta_\tau)\frac{1}{\sqrt{m}}\Theta(\beta_\tau)+o_p(1).$$

Therefore $\sqrt{m}(\tilde{\beta}_\tau-\beta_\tau) \to N(0, G^{-1}(\beta_\tau)V\{G^{-1}(\beta_\tau)\}^T)$ as $m \to +\infty$. □
